# The online purchase of medicines – an international pharmacists’ perspective

**DOI:** 10.3389/fphar.2025.1625826

**Published:** 2025-10-06

**Authors:** Tomasz Zaprutko, Krzysztof Kus, Yuliia Kremin, Bohdan Hromovyk, Hristina Lebanova, Dušanka Miloša Krajnović, Ivana Stević, Emilija Simić, Kateryna Dorykevych, Julia Cynar, Wiktor Eliasz, Afonso Miguel Cavaco

**Affiliations:** ^1^ Department of Pharmacoeconomics and Social Pharmacy, Poznan University of Medical Sciences, Poznań, Poland; ^2^ Department of Organization and Economics of Pharmacy, Danylo Halytsky Lviv National Medical University, Lviv, Ukraine; ^3^ Department of Pharmaceutical Sciences and Social Pharmacy, Medical University Pleven, Pleven, Bulgaria; ^4^ Department of Social Pharmacy and Pharmaceutical Legislation, Belgrade University, Beograd, Serbia; ^5^ Postgraduation Programe of the Pharmaceutical Management and Marketing at the Faculty of Pharmacy, University of Belgrade, Beograd, Serbia; ^6^ Poznan University of Medical Sciences, Doctoral School, Department of Pharmacoeconomics and Social Pharmacy, Poznan University of Medical Sciences, Poznań, Poland; ^7^ Student Scientific Society, Department of Pharmacoeconomics and Social Pharmacy, Poznan University of Medical Sciences, Poznań, Poland; ^8^ Faculty of Pharmacy, University of Lisbon, Lisboa, Portugal

**Keywords:** medicines, online access, affordability and accessibility of medicines, pharmacists, questionnaire

## Abstract

**Background:**

Accessibility and affordability of medicines are key for patients’ effective treatment. However, drug prices are soaring, and patients are looking for cheaper medications in Europe and beyond. This study aimed to evaluate European pharmacists’ perceptions and attitudes about the impact of global inflation and the military conflict setting in Ukraine on patients’ buying medicines behaviours. A secondary objective comprised the problem of buying medicines from illegal online sources like social media or non-regulated marketplaces.

**Methods:**

An observational cross-sectional study was conducted from April 2023 to April 2024 using an anonymous and self-designed questionnaire consisting of 11 questions. The survey was created using Google Forms. The survey link was shared mostly by e-mail amongst practising pharmacists from five European countries.

**Results:**

Events of the recent years, such as inflation, the COVID-19 pandemic, or the war in Ukraine were perceived by the pharmacists as contributing to patients looking for better medicines prices, mostly related to online purchases. The most important factors influencing online purchase of medicines beyond price were convenience of shopping anytime, and fast and free delivery. There is a perceived growing interest in buying medicines from online facilities other than e-pharmacies, such as local websites, social media or global marketplaces (e.g., aliexpress.com). Pharmacists do not actively inform their patients about the possible risks of buying medicines from unverified online sources.

**Conclusion:**

International disruptions seem to contribute to patients’ looking for better prices of medicines. There is a need for societal education about the risks related to the possibility of counterfeited medicines online, as well as pharmacists’ training to prioritize patient information and counselling on the hazards of medication acquisition from non e-pharmacies. There is an urgent need for global amendments to the pharmaceutical law to protect patients from illegal e-sources of medicines while keeping in-person services and offline purchases.

## Introduction

Affordability and accessibility of medicines are crucial for patients’ quality of life and their effective treatment. Despite this, one-third of the global population cannot access medicines, and the prices of medicines are one of the main reasons for this (Vogler et al., 2015).

Before the COVID-19 pandemic, the majority of the population in France (90%) or Denmark (84%) declared that buying medicines had no repercussions on the household budget. However, in countries like Poland (40%), Cyprus (34%), and Bulgaria (33%), the trend was reversed and the cost of medicines was a heavy financial burden for household expenditures (The burden of healthcare on households’ budgets, 2025).

Nowadays, drug prices are soaring globally. Thus, countries and individuals spend more on medicines (POLITICO, 2024). For instance, in the US the cost of specialty drugs increased of 43% in 2021 since 2016 (sdp-trends-prescription-drug-spending.pdf, 2024). In Belgium, however, drug spending increased 9% between 2021 and 2022. In France it was 8.5% in 2023 compared to 2022 (POLITICO, 2024). Medicines-related expenses are a leading part of healthcare expenditures and have been increasing even faster than other healthcare expenditures (Bertoldi et al., 2011). The increase might result from several factors including ageing societies, demography or the access to innovative and frequently pricy technologies (POLITICO, 2024; Bertoldi et al., 2011). Additionally, circumstances such as public health threats (e.g., COVID-19 pandemic), international disruptions (e.g., the war in Ukraine), supply chain constraints (e.g., increasing costs of pharmaceutical ingredients), and other economic disturbances contributed to the rising global inflation rate, which is one of the main reasons for the rapidly increasing prices of medicines (Raven et al., 2022; Zaprutko et al., 2024).

In some countries, the costs of medicines were rising faster than the inflation rate (Raven et al., 2022; Cubanski and Published, 2022) while in the others the growth was equal, but still, the rate was high (Yeung et al., 2022; Erker, 2024). This may contribute to medications that will not be accessible to a growing number of people, especially those living in poverty (Raven et al., 2022; Inflation’s Impact on the Pharmaceutical Industry, 2024; economy, 2024). Considering that access to essential medicines is a crucial human right (Vogler et al., 2015) allowing them to complete their healthcare needs (Almomani et al., 2023a), it is unsurprising that patients are looking for several ways to get their medication at lower prices.

One of the options for searching for cheaper offers is online shopping (Limbu and Huhmann, 2024). The phenomenon is due to a broader range of access to offers, convenience, the possibility of comparing prices, or the guarantee of access to goods. However, studies show (Almomani et al., 2023a; Almomani et al., 2023b) that up to 90% of online drug purchases are associated with the purchase of substandard, counterfeit, or falsified products. Despite this, the number of people purchasing medicines online is constantly growing (Almomani et al., 2023a; Limbu and Huhmann, 2024), with a marked increase in medicines from social media, encrypted messaging applications, and even marketplaces like AliExpress (Almomani et al., 2023a; Zaprutko et al., 2022; Shaping Europe’s digital future, 2023).

Considering the rapidly growing online purchase of medicines also affecting the EU region, which is also facing military disruption, the study aimed to evaluate pharmacists’ attitudes and perceptions about the impact of global economic disturbances (e.g., inflation) and other disruptions like the war in Ukraine on patients’ behaviours toward buying medicines. It was also intended to assess the pharmacists’ judgments about patients’ search for and buying medicines from illegal online sources like social media or Asian marketplaces.

## Materials and methods

Authors of this study cooperate within the scientific group Pharmacy of Tomorrow (PoT). Thus, the awareness of the several problems within the pharmaceutical markets, which are discussed during meetings and when planning common studies. Considering the rapidly growing online purchase of medicines, the study was to survey pharmacists from three European Union (EU) countries (Portugal (PT), Poland (PL), Bulgaria (BG)) and two countries outside the EU (Serbia (RS), and Ukraine (UA)). The countries’ selection was to get a cross-sectional view of well-developed pharmaceutical services through the differences in the local economy, policy, and war reality (UA).

The study followed a cross-sectional design and was conducted from April 2023 to April 2024 by the several project partners. The data collection instrument was an anonymous and self-developed questionnaire consisting of 11 questions (10 single-choice and 1 multiple-choice). The main questionnaire (Supplementary Table S1) was preceded by a short sociodemographic section (Supplementary Table S2) related to the characteristics of the respondents (5 single-choice questions concerning, e.g., gender, age) and the frequency of Internet use and online shopping (2 single-choice questions). The facets related to participants’ Internet use habits were to provide the background, allowing the assessment of whether pharmacists participating in the study may feel comfortable with the topic of the online purchase of medicines.

The study tool was based on the literature search about online medication shopping (Almomani et al., 2023a; Limbu and Huhmann, 2024; Almomani et al., 2023b; Fittler et al., 2024; Moureaud et al., 2021) and our experience gained from the pharmacy practice. An initial English version of the questionnaire was created and shared for peer adequacy, completeness and face validation. Then, we translated English version into the local languages using two different translation resources (DeepL and Google) supervised by each team member, providing a clear study tool for participants from individual countries. Very few local adjustments were made, except in the Serbian version, where the 2nd and 3rd questions from the main body of the study tool were rewritten because both Over-the-counter (OTC) and prescription (Rx) online medication purchases were forbidden by the legislation in Serbia during the study data collection. The survey was created using Google Form and shared within the online professional groups (in Bulgaria) via a survey link sent mainly by e-mail, within an invitation message to participate in the study. The email was sent to publicly available pharmacy email addresses from each country. The invitation dissemination also resulted from the team members cooperation and membership in local pharmacy chambers or associations. Using the information received, each pharmacy was marked numerically for anonymization.

The target participants for this study were pharmacists. Pharmacy technicians (PTs) were not considered since the role of PTs and their professional qualifications are not the same in the countries participating in the study. Participation in the survey was voluntary, and no incentives were offered. In case of a lack of response to our e-mail, we repeated the request 2 weeks after. Lack of response after this time was considered a refusal to participate in the study. Although an online survey seems more straightforward or comfortable for the respondents and researchers, we also delivered the printed questionnaires to pharmacies upon request, through the local partners. However, the majority of the questionnaires distributed were online since respondents would prefer to participate anytime or anywhere. Some participants asked for the link, even getting the paper version of the questionnaire, so no difference from the electronic or paper was taken into account. Participation may have also happen from pharmacists sharing the link, e.g., within the pharmacy chain and internal groups like WhatsApp, for instance. The main goal was to have completed questionnaires and no sample power calculations or statistical representation were aimed. The method of participation request distribution impeded the collection of information about the real number of questionnaires delivered and, as a result, the response rate. Besides, information gained directly from the pharmacy was marked numerically for anonymization. It prevented the presentation of, e.g., the pharmacy’s name and, consequently, the acquisition of advertising pharmacies, which is forbidden in Poland. In the case of Ukraine being under war pressure, it was not easy to reach some of the pharmacists directly. Despite the limitations, the data collection was unified.

Although the data collection starting date (15 April 2023) was the same for all participants, as well as a common due date (15 October 2023), more time was needed in Poland and Portugal due to the unsatisfactory number of response, after the two send outs. We obtained 372 responses from Ukraine, followed by Serbia with 244 responses. In Poland and Bulgaria 213 and 83 questionnaires were completed, respectively. We obtained 62 answers from Portugal.

The study was subjected to the opinion of the Ethics Committee, which stated (decision KB-903/22) that this research is not a medical experiment and, according to Polish law and GCP regulations, does not require the approval of the Bioethics Committee.

### Statistical analysis

Nominal data were analyzed using the chi-square test of independence. All tests were considered significant at *p* < 0.05. The Test of Proportions and the Conover *post hoc* test determined significant differences between group percentage results.

## Results

In each country, the females participating in the study had a statistically significant (p < 0.05) dominance. Study participants represented primarily those between 26 and 40 years of age, while pharmacists in the age group above 60 were in minority (3.70%) in individual countries. The average work experience was the lowest in Bulgaria (7.73 years) and the highest in Serbia (12.66 years). Study participants represented primarily cities, which were frequently academic centres. From Portugal, however, we obtained the highest number of answers from those who worked in towns (54.39%).

Pharmacists’ Internet and online shopping habits showed a clear majority of daily Internet users. In BG and PT 100% of respondents were daily Internet users. In RS and UA it was 99%. Nonetheless, in PL there was a statistical significance (p < 0.0001) related to the occasional (5.63%) and daily (94.37%) Internet use. In the case of online shopping, Polish, Ukrainian, and Bulgarian respondents most frequently indicated “a few times a month,” whereas in Portugal and Serbia, it was “a few times a year” for online shopping. We found a statistical significance (p < 0.05) between the lowest and the highest values in PT, RS, and UA, as well as related to the answer options.

We found (p < 0.001) significant differences in the opinion of study respondents concerning global inflation and geopolitical disturbances (like the war in Ukraine) contributing to patients looking for better prices of medicines. Unsurprisingly, we obtained the highest number of affirmative answers (93.30%) from Ukraine, which is at war. On the contrary, the lowest (78.57%) affirmative answers were from Portugal compared to, e.g., Poland or Bulgaria with 86.85% and 81.90% respectively.

Pharmacists from Poland (57.28%) stated that due to the inflation rate, their patients have been considering buying OTCs from online pharmacies. In Serbia, pharmacists stated (55.33%) that in their opinion, patients would consider shopping OTCs in online pharmacies if it were legally allowed. On the contrary, in Bulgaria (53.00%), Ukraine (50.30%), and Portugal (35.71%), the most prevalent answer was that patients still prefer traditional purchases in pharmacies. The same question was asked concerning Rx medicines. In that case, the answer indicating that patients still prefer traditional purchases was the first choice in each country. In Bulgaria and Serbia, such a service is not accessible. However, we asked Serbian pharmacists if that was possible they believe that more than 55% of patients would consider even partial Rx shopping in online pharmacies. It is in line with results from Poland, where such a service is available (click and collect in pharmacy), and 30.05% of Polish pharmacists believe that their patients consider buying Rx medicines from online pharmacies, even if partially.

**FIGURE 1:**
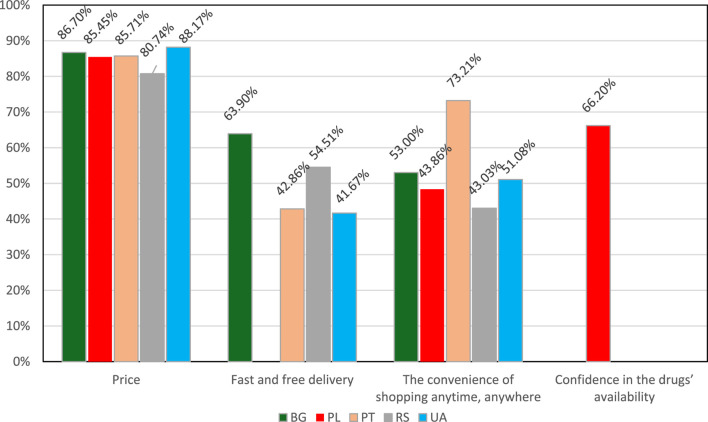
The most popular criteria for buying medicines online. BG–Bulgaria, PL–Poland, PT–Portugal, RS–Serbia, UA–Ukraine. *Serbia–if it would be legally allowed to buy medicines online.

Unsurprisingly, price is the main reason for buying medicines from online pharmacies in each country. “The convenience of shopping anytime, anywhere” and “fast and free delivery” were the following answers for all countries apart from Poland, where “confidence in the drugs’ availability” was a frequently chosen option. The distribution of the three most frequent answers is presented in Figure 1.

**FIGURE 2:**
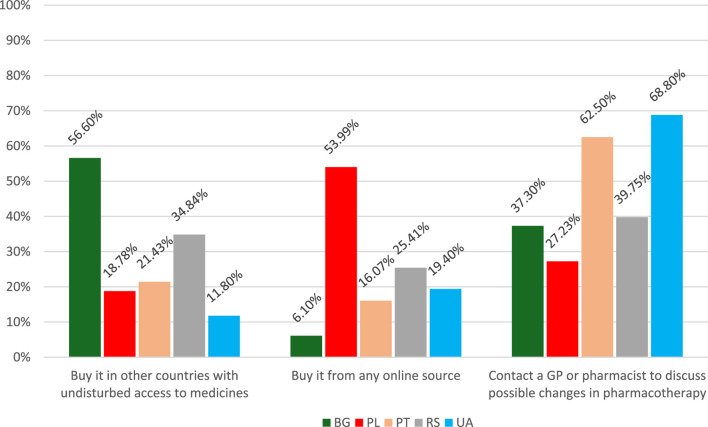
Sources of access to medicines in the case of drug shortages. BG–Bulgaria, PL–Poland, PT–Portugal, RS–Serbia, UA–Ukraine. *Serbia–if it would be legally allowed to buy medicines online.

The search and buying of medicines online might result from drug shortages. In this case, the answers distribution is presented in Figure 2.

**FIGURE 3:**
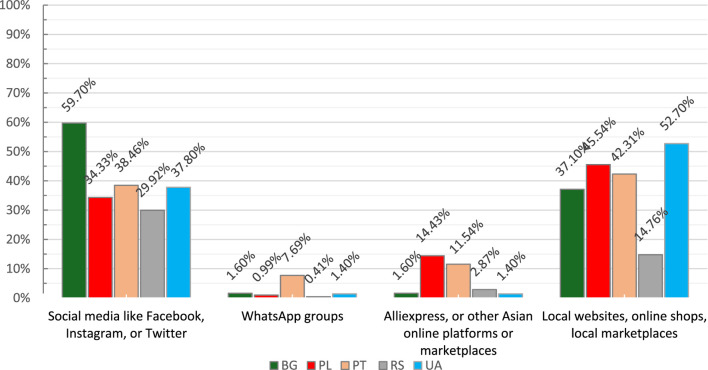
The alternative online sources of access to medicines. BG–Bulgaria, PL–Poland, PT–Portugal, RS–Serbia, UA–Ukraine.

Pharmacists were asked if they thought their patients had ever looked for or bought pharmaceutics from other online sources than online pharmacies. The affirmative answer was the leading one in Bulgaria (63.90%), Poland (57.75%), Serbia (48.36%), and Portugal (46.43%). In Ukraine, the first choice was “I am not sure,” (46.2%) which was the second most frequent response in previously mentioned countries. “No” was the rarest answer in each country. In the case of an affirmative answer to this question, participants were asked to provide their opinion about the possible source of access to such goods. In Bulgaria and Serbia, “social media like Facebook, Instagram or Twitter” was the most frequent answer, followed by “local websites, online shops, local marketplaces”. In other countries, the distribution of responses was the opposite. Interestingly, pharmacists from Poland and Portugal (14.43% and 11.54%, respectively) also indicated “AliExpress, or other Asian online platforms or marketplaces” as a third option for access to medicines. The answers distribution is presented in Figure 3.

**FIGURE 4:**
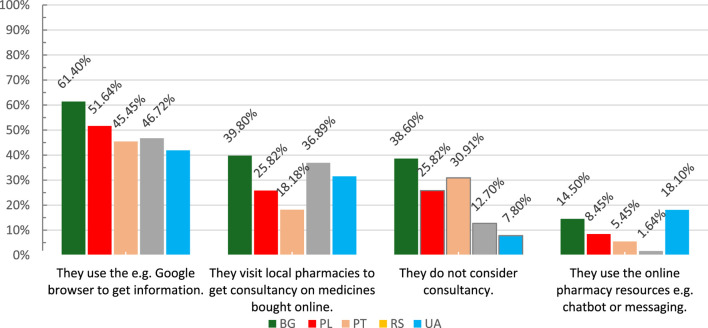
The sources of consultancy services during online shopping. BG–Bulgaria, PL–Poland, PT–Portugal, RS–Serbia, UA–Ukraine.

Regardless of the country, pharmacists participating in the study assumed that patients do not verify the legality of the online source before the purchase. However, it may happen but if the online source is unknown to them. Pharmacists also claim that patients, if needed during online shopping, choose Google Browser as a leading source of health information. The answers distribution is presented in Figure 4.

Our study revealed that information about the possible dangers of buying medicines from unverified online sources was not provided in many cases, with 32.86% of answers in Poland followed by Bulgaria (26.50%), and Ukraine (22.6%). The answer indicating that such information is provided very rarely was the most popular in Portugal (58.91%), Poland (46.48%), and Ukraine (32.8%). In Serbia (33.20%) and Bulgaria (28.9%) the answer “Yes, maybe a couple of times during a month” was the most common.

Finally, we asked study participants if online pharmaceutical purchases should work as “click & collect,” which means online order and collection in person. The answer “yes, it is a good idea if products are collected in the pharmacy or home delivered by the pharmacy” was most popular in Poland (62.44%), Portugal (52,73%), and Ukraine (45.20%), followed by the answer indicating the preference of usual pharmacy service. In Bulgaria and Serbia, the distribution was the opposite. Nonetheless, there was a statistical significance (p < 0.0001) between the lowest and the highest values in the sampled countries and related to the above answer options. In our study, the study pharmacists did not think that online pharmaceutical purchases with vending machines and Uber or Bolt home delivery were a good idea.

## Discussion

To the best of the authors’ knowledge, this was the first international study aimed at social pharmacy facets related to online shopping of medicines including the possible impact of the inflation rate and war in Ukraine. The most important findings of our study were that disturbances of recent years, led by, e.g., inflation, the COVID-19 pandemic, or the war in Ukraine, contribute to pharmacists’ opinions, indicating that these factors contribute to the growing phenomenon of looking for better prices of medicines, which may also result in more frequent online shopping for medicines. As in other studies (Limbu and Huhmann, 2024; Almomani et al., 2023b; Fittler et al., 2024) we found that price, convenience of shopping anytime, and fast and free delivery are the most important selection criteria when buying medicines online. We also revealed that there is a trend of buying medicines in other online facilities than online pharmacies. These are not only local websites, but social media and Asian marketplaces are also indicated as sources of uncontrolled access to medicines. Despite the pharmacist’s awareness of the growing impact of getting medicines from these online sources, information about the possible dangers of buying pharmaceuticals from unverified online sources is not an everyday practice for most pharmacists participating in this study.

Pharmacists are the most accessible healthcare professionals (Zaprut et al., 2024; Steltenpohl et al., 2018), so their role in providing medicines, healthcare services, and education is indisputable and key for public health. This aligns with our study, where in Bulgaria, Ukraine, and Portugal, study participants claimed that patients still prefer traditional purchases in pharmacies particularly in the case of Rx medicines. However, we found a growing interest in buying OTCs from online pharmacies in Poland and Serbia (if allowed) and a higher level of acceptance in the case of online trade of Rx brands in these countries. This was also observed in the trend revealed by Fittler et al. (2024), who noticed a significant increase in the turnover of medicines from online pharmacies in Central European countries. The search for better medicine prices might also result from the inflation rate. According to the Eurostat, the inflation rate was very high globally in 2022 reaching, e.g., in the EU, a historical high of 9.2% (Consumer prices - inflation, 2024), but in 2023 has started to unevenly decrease, revealing differences between countries (Consumer prices - inflation, 2024). Hence, according to Eurostat, it was 12.1% and 10.9% in Serbia and Poland, respectively. For comparison, in Bulgaria and Portugal, it was 8.6% and 5.3% (Moureaud et al., 2021). In Ukraine, which is under war pressure, it was 20.18% in 2022, and in 2023, it declined to 12.85% (Ukraine Inflation Rate, 2025). It seems that among the reasons already mentioned in the paper for the inflation rate, the COVID-19 pandemic is the most influential worldwide (Faramarzi et al., 2024). A lot of countries, even rich ones, still struggle with the effects of pandemics (Faramarzi et al., 2024; Fasanya et al., 2023; Gharehgozli and Lee, 2022). It refers to economic disturbances, including the bankruptcy of several companies or employee layoffs (Faramarzi et al., 2024). It may result from the fact that, according to Gharehgozli and Lee (2022), the high inflation after COVID-19 is not transitory but is persistent.

Regardless of the country, pharmacists participating in our study indicated that their patients prefer the traditional pharmacy services over online forms. This includes hybrid services, where products are collected in the pharmacy or are delivered by the pharmacy at home. This service design accelerated during the COVID-19 pandemic. Nowadays, online pharmacies are an integral part of healthcare provisions in developed countries (Fittler et al., 2024; Fittler et al., 2022). However, Hock et al. (Product E-Commerc - Szukaj w Google, 2024) in the study “Regulating online pharmacies and Medicinal Product E-Commerce” revealed that about 66% of countries do not have regulations for the online trading of medicines. Besides, according to the Alliance for Safe Online Pharmacy, around 95% of the 35000 active online pharmacies work illegally (Almomani et al., 2023a). These facilities sell counterfeit, fake, or unapproved drugs (Limbu and Huhmann, 2024; Almomani et al., 2023b). Thus, the “click&collect” model of e-pharmacy seems to be desired for the pharmaceutical market. It might be a temporary solution that allows for incorporating needed regulations and law amendments globally. Nonethelss, the regulation of the turnover of prescription-only medicines might be more challenging than in the case of OTC medicines. However, Rx medicines picked up in the traditional pharmacy might be free of charge, like in Scotland or Wales (Almomani et al., 2023b). Thus, buying such medicines online would be simply unprofitable. It confirms the need for active societal education, especially considering the rapid growth of the online pharmacy market (Limbu and Huhmann, 2024). There is also a need for engagement of healthcare decision-makers, media, scientists, and health professionals led by pharmacists working directly with medicines and patients. However, the results of our study revealed that pharmacists’ engagement in informing patients about the possible risks of buying pharmaceuticals from unverified online sources is insufficient, being relatively rare and irregular.

We revealed that in pharmacists opinion patients do not verify the lagality of the online source before the purchase. It may happen only if the online source is unknown. These findings might partially explain how around 95% of online pharmacies may operate illegally (Almomani et al., 2023a). The trust in several unverified online sources of access to medicines contributes to the fact that several marketplaces, including social media, have become a space of medicine turnover (Zaprutko et al., 2022; Moureaud et al., 2021). Besides, for many people, the Internet, including social media, is the first and most trustworthy source of health information (Song et al., 2016; Coloma et al., 2015). It seems to be an even stronger trend after the COVID-19 pandemic, which boosted the omnipresent applications and other online solutions surrounding us in everyday life.

Apart from the share of online pharmacies operating illegitimately (Almomani et al., 2023a) there is another challenge in the field. These facilities frequently offer prescription-only medicines without a prescription and consultation (Almomani et al., 2023a; Fittler et al., 2024). This phenomenon was also confirmed by Novak et al. (2016) in a study including 5 European countries where authors revealed that individuals could reach stimulants or even opioids without a prescription. The uncontrolled access to several medicines is at risk of rapid development (Zaprutko et al., 2022; Moureaud et al., 2021). Although pharmacists participating in our study claimed that in the case of prescription-only medicines, there is still a predominance of traditional pharmacy services, they also indicated that, in their opinion, patients looked for/bought pharmaceutics in other online facilities than online pharmacies. The findings point to local websites, social media, and Asian marketplaces like aliexpress.com, for instance. This is consistent with the study by Li et al. (2019) who presented that social media like Facebook or Instagram were the places for drug dealing and opioid sales. Zaprutko et al. (2022) also found Facebook as a source of uncontrolled access to medicines, with the majority of prescription-only brands with morphine, antipsychotics, or oncology medicines offered amidst other substances or therapeutic groups. Among several possible reasons for such a situation, drug shortages (Almomani et al., 2023a; Zaprutko et al., 2020) are leading people to search for alternative sources of medicines. It is also related to the confidence in drug availability and 24/7 accessibility with simultaneous lower costs contributing to general convenience, which compensates the risks of getting medicines from unverified sellers (Almomani et al., 2023a; Limbu and Huhmann, 2024). The present findings support that fast and free delivery was also essential to purchasing online medicine.

Although in developed countries, online pharmacies must be registered with competent authorities (Almomani et al., 2023a; Moureaud et al., 2021) presented that people in 29 states in the US died due to the unintentional use of falsified analgesics or alprazolam. In the United Kingdom, however, 1 in 10 people bought falsified medical products online in 2020 (Almomani et al., 2023a). These findings, both with our results, confirm the urgent need for international cooperation and harmonization of regulations aimed at patient protection. All stakeholders are required to find a balance between convenience and safety. Hence, they should address the challenges of the online pharmacy market and its safety by maximizing the benefits of legal online pharmacies (Fittler et al., 2024).

## Limitations

Despite the importance of the topic, the study has some limitations. The sample could be higher in individual countries and aimed at statistical representativeness. Nonetheless, we offered no incentives to study participants. Considering many questionnaire queries, pharmacists might not be interested in filling out a study tool without some incentives. Besides, the difference in the number of received responses might result from, e.g., different numbers of inhabitants in the analyzed countries. Nonetheless, RS is a country with a similar number of inhabitants to BG and a lower number than PT. Despite this, we obtained more answers from RS than from BG and PT. Hence, the differences might also result from local community characteristics relating to willingness to participate in such studies and market characteristics, for instance.

Moreover, due to the method of participation request distribution and differences in data collection reality (e.g., war in UA), it was impossible to get information about the exact number of pharmacists who received the request and consequently to present the response rate. Although it would be interesting to analyze results according to sociodemographic characteristics, the analysis might not be valuable because of the small subgroups in some analyzed countries (e.g., PT or BG). To mitigate the limitation of study sampling, we used non-parametric tests aimed at the consistency of results. We also refrained from overinterpreting marginal or country-specific effects, especially in cases where the sample size was too limited to support strong claims. However, further bootstrap resampling or subsampling within the larger sample to match smaller group sizes might have helped with comparisons. Hence, further studies are needed to confirm the trend of the presented results.

It is also worth noticing that solely pharmacists were the target group in this study. Including PTs could increase the number of received answers in all countries. However, the role of PTs and their professional qualifications differ in the countries participating in the study. Thus, they were not considered in the study.

Moreover, the study did not demonstrate both translations and some cultural equivalency. Another limitation is related to the questionnaire, which was face-validated. The study might benefit if we ask patients and pharmacists for their opinions concerning the potential impact of free-of-charge prescription-only medicines if they are obtained from regular pharmacies. Besides, it could be valuable to develop the last but one question (number 10 in Supplementary Table S1) and assess if and how often patients raise the possible dangers of buying pharmaceuticals from unverified online sources during visits to the pharmacy. It could present social awareness about the phenomenon of online purchase of medicines. However, it might be considered a separate topic.

We are also aware that in several countries, the trend of online medicine purchases and the demand for cheaper medicines may vary, influenced by different factors. Nevertheless, despite these differences, recent circumstances such as inflation, the COVID-19 pandemic, and the war in Ukraine have been perceived as contributing to patients seeking better prices for medicines, primarily through online purchases.Furthermore, although the current study reveals the trend of the obtained results, further studies are needed to develop the samples and results, and complete conclusions despite the importance of the study.

## Conclusion

In recent years, a range of significant factors - most notably inflation, the COVID-19 pandemic, and the war in Ukraine affecting not only Europe, have contributed to patients seeking better prices for medicines. It pushes patients to purchase medicines online, especially OTC medicines. However, the interest in online access to prescription-only medicines is a developing trend. According to the obtained pharmacists’ opinions, the study confirms the phenomenon of patients buying or looking for medicines on other online facilities rather than official e-pharmacies. These are social media and marketplaces like aliexpress.com. However, there is still space for in-person services and offline purchases of medicines. We are aware that the researched topic is complex, and several facets must be included. Nevertheless, market changes are inevitable and should be considered urgently to provide present-day access to medicines with simultaneous patient protection. It would also lead to the saving of resources we spend to treat the side effects of falsified medicines or to cover the presenteeism or absenteeism of an employee or caregiver after using falsified medication.

## Data Availability

The original contributions presented in the study are included in the article/Supplementary Material, further inquiries can be directed to the corresponding author.
