# Supplementary material for: The online purchase of medicines – an international pharmacists’ perspective
Source: Front Pharmacol. 2025 Oct 6;16:1625826. doi: 10.3389/fphar.2025.1625826 (PMC12536223; doi:10.3389/fphar.2025.1625826)
Supplement: Supplementary file 1 [file Table1.docx]

Table 1 (supplementary). Questions content and possible answers.

| Question | Question content | Possible answers |
| --- | --- | --- |
| 1 | In your opinion, do global (e.g., inflation) economic and geopolitical disturbances (e.g., the war in Ukraine) contribute to your patients looking for better prices of medicines and pharmaceutical products? | - Yes - No - I have no opinion |
| 2 | In your opinion, do your patients consider to move from buying OTCs (without prescription) pharmaceuticals to online pharmacies shopping? | - Yes, they have already moved with all possible shopping to online pharmacies - Yes, they consider even partial shopping in online pharmacies - Yes, but they don’t care if is it a legal online pharmacy or other e-commerce - No, they prefer traditional purchases in pharmacies |
| 3 | In your opinion, do your patients consider to move from buying Rx (prescription medicines) pharmaceuticals to online pharmacies shopping? | - Yes, they have already moved with all possible shopping to online pharmacies - Yes, they make partial purchases in legal online pharmacies using electronic prescriptions in accordance with the procedure established by the Ministry of Health of Ukraine - No, such a service is not available in my country (due to local regulations) - No, they prefer traditional purchases in pharmacies |
| 4^^^ | In your opinion, what is/would be selection criterion for your patients when buying medicines from an online pharmacy? *(up to 3 answers possible*) | - Price - Fast and free delivery - The convenience of shopping anytime, anywhere - Confidence in the drugs’ availability - Transaction security - The reputation of online pharmacy - Reviews of friends or relatives about a particular online pharmacy - Easy site navigation/layout |
| 5 | In your opinion, what would your patients do in the case of medicines shortages? | - Buy it in other countries with undisturbed access to medicines - Buy it from any online source - Contact a GP or pharmacist to discuss possible changes in pharmacotherapy |
| 6 | Do you think your patients ever looked for/bought pharmaceutics in other online facilities than online pharmacies? | - Yes - No - I am not sure |
| 7 | Considering the affirmative answer to the previous question, please indicate the source | - Social media like Facebook, Instagram, or Twitter - WhatsApp groups - Alliexpress, or other Asian online platforms or marketplaces - Local websites, online shops, local marketplaces |
| 8 | In your opinion, do your patients verify the legality of the online source before the purchase? | - Yes, always - Yes, but only in the case of new or unknown online sources - No |
| 9 | In your opinion, where and how do your patients get consultancy services during online shopping? | - They use the online pharmacy resources e.g. chatbot or messaging - They use the e.g. google browser to get information - They visit local pharmacies to get consultancy on medicines bought online - They do not consider consultancy |
| 10 | Have you ever informed your patients about the possible dangers of buying pharmaceuticals from unverified online sources? | - Yes, frequently (from everyday to at least once a week) - Yes, maybe a couple of times during a month - Yes, but very rarely (about once in three or six months) - No |
| 11 | In your opinion, do you think online pharmaceutical purchases should work as “click&collect” (online order and local collection)? | - Yes, it’s a good idea if products are collected in the pharmacy or home delivered by the pharmacy - Yes, it's a good idea even with vending machines or home delivery (e.g., Uber, Bolt) - No, I prefer usual pharmacy service |

^^^ multiple choice possibility
