# Supplementary material for: The online purchase of medicines – an international pharmacists’ perspective
Source: Front Pharmacol. 2025 Oct 6;16:1625826. doi: 10.3389/fphar.2025.1625826 (PMC12536223; doi:10.3389/fphar.2025.1625826)
Supplement: Supplementary file 2 [file Table2.docx]

Table 2 (supplementary). Questions about respondent characteristics.

| Question | Question content | Possible answers |
| --- | --- | --- |
| 1 | Gender | - Female - Male |
| 2 | Age | - 18-25 years - 26-40 years - 41-59 years - > 60 years |
| 3 | Country | multiple choice possibility |
| 4 | How many years do you work in community pharmacy? | multiple choice possibility |
| 5 | Place of work | - city (e.g., with an University) - small city - town - village |
| 6 | Frequency of Internet use | - daily, - weekly - from time to time - never |
| 7 | Frequency of online shopping? | - regularly (almost everyday to a few times a week) - a few times a month - a few times a year - never |
